# Dysfunction in Serotonergic and Noradrenergic Systems and Somatic Symptoms in Psychiatric Disorders

**DOI:** 10.3389/fpsyt.2019.00286

**Published:** 2019-05-21

**Authors:** Yi Liu, Jingping Zhao, Xiaoduo Fan, Wenbin Guo

**Affiliations:** ^1^Department of Psychiatry, The Second Xiangya Hospital of Central South University, Changsha, China; ^2^National Clinical Research Center on Mental Disorders, Changsha, China; ^3^University of Massachusetts Medical School, UMass Memorial Medical Center, Worcester, MA, United States

**Keywords:** somatic symptoms, mono-aminergic neurotransmitters, norepinephrine (NE), serotonin (5-HT), pathophysiology

## Abstract

Somatic symptoms include a range of physical experiences, such as pain, muscle tension, body shaking, difficulty in breathing, heart palpitation, blushing, fatigue, and sweating. Somatic symptoms are common in major depressive disorder (MDD), anxiety disorders, and some other psychiatric disorders. However, the etiology of somatic symptoms remains unclear. Somatic symptoms could be a response to emotional distress in patients with those psychiatric conditions. Increasing evidence supports the role of aberrant serotoninergic and noradrenergic neurotransmission in somatic symptoms. The physiological alterations underlying diminished serotonin (5-HT) and norepinephrine (NE) signaling may contribute to impaired signal transduction, reduced 5-HT, or NE release from terminals of presynaptic neurons, and result in alternations in function and/or number of receptors and changes in intracellular signal processing. Multiple resources of data support each of these mechanisms. Animal models have shown physiological responses, similar to somatic symptoms seen in psychiatric patients, after manipulations of 5-HT and NE neurotransmission. Human genetic studies have identified many single-nucleotide polymorphisms risk loci associated with somatic symptoms. Several neuroimaging findings support that somatic symptoms are possibly associated with a state of reduced receptor binding. This narrative literature review aimed to discuss the involvement of serotonergic and noradrenergic systems in the pathophysiology of somatic symptoms. Future research combining neuroimaging techniques and genetic analysis to further elucidate the biological mechanisms of somatic symptoms and to develop novel treatment strategies is needed.

Somatic symptoms in psychiatric disorders are symptoms that have persistent bodily complaints but have found no explanatory structural, organic causes, or other specified pathology after a sufficient physical examination or investigation by physician or healthcare providers (1). The most common somatic symptoms include musculoskeletal pain, abdominal pain, fatigue, dizziness, ear, nose, throat symptoms and gastrointestinal symptoms. Somatic symptoms, including a range of physical symptoms, such as pain (e.g., stomachache, headache, and neuropathy), muscle tension, body shaking, difficulty breathing, heart palpitation, fatigue, and gastrointestinal symptoms, are commonly seen in individuals with major depressive disorder (MDD), anxiety disorders, and other psychiatric disorders (2). About 76% of patients diagnosed with depression had somatic symptoms, such as back pain, headache, stomach pain, migraine, and neuropathic pain (3, 4). Severity of depression is positively associated with the frequency and severity of somatic symptoms (5, 6). Somatic symptoms were able to predict subsequent self-reported symptoms of depress in women patients with MDD (7). Somatic symptoms are also a common feature of anxiety disorders (8). Patients suffering from anxiety disorders often have somatic complaints, such as feeling jittery, muscular tension, stomachache, headache, and sweating (9). Young patients with anxiety disorders are more likely to report somatic symptoms than their healthy peers (10). Moreover, studies have found that somatic symptoms are related to acute stress disorder, posttraumatic stress disorder, and personality disorders (11).

A heightened awareness of certain body sensations may trigger somatic symptoms (12). Somatic symptoms may be a mechanism through which patients with depression or anxiety react to their emotional distress (13). Childhood neglect and adversity, childhood abuse, chaotic lifestyle, stress, alcohol abuse, and substance abuse are the risk factors for somatic symptoms. Women more likely present somatic symptoms than men. Antidepressant drugs, including selective serotonin (5-HT) reuptake inhibitors (SSRIs), dual 5-HT and norepinephrine (NE) reuptake inhibitors (SNRIs), and tricyclics are effective in treating somatic syndromes. The therapeutic effects of these drugs may be due to their effects on the 5-HT and NE systems. Hence, abnormal serotonergic and noradrenergic systems, which are indicated by low level of monoamine neurotransmitters, reduced production and/or release, pre- and/or post-synaptic receptor dysfunction, excessive self-inhibition, and decreased excitatory inputs, may play a predominant role in the pathophysiology of somatic symptoms. This narrative review paper was to summarize the role of serotonergic and noradrenergic systems in the development somatic symptoms.

## 5-HT and NE Neurotransmitter Systems

5-HT is a monoamine neurotransmitter. Serotonergic neurons exist mainly in the dorsal and median raphe nuclei in the brainstem. 5-HT is released into the extracellular space from presynaptic nerve terminal, and is cleared primarily by neurotransmitter uptake, mediated by the 5-HT transporter. 5-HT receptors contain presynaptic autoreceptors and postsynaptic receptors. The 5-HT autoreceptors are key factors in the self-inhibitory mechanism of serotonergic neuronal activity. Activation of inhibitory 5-HT autoreceptors regulates 5-HT neuronal firing and maintains the homeostasis of the serotonergic system. 5-HT exerts its effects through its interaction with 5-HT receptors, including the 5-HT1 to 5-HT7 families, some of which have several subtypes (14, 15). The effects of 5-HT depend on the cell type and subtype of the receptor it acts on. A growing body of evidence has suggested the role of the serotonergic system in somatic symptoms. Most previous studies focused on 5-HT1, 5-HT2A, 5-HT2C, 5-HT3, 5-HT4, and 5-HT7 receptors.

NE is also a monoamine neurotransmitter in the brain. The primary source of NE is neurons in the locus coeruleus, which is situated at the floor of the fourth ventricle in the pontine brain (16). Other noradrenergic neurons include nuclei of the lateral tegmentum and the solitary tract. NE released from the locus coeruleus regulates brain function through various ways. The locus coeruleus receives afferent projections from the various brain regions, such as the insular cortex, the hypothalamus, the central amygdala, and the cerebral cortex (17–20). Adrenoceptors can be classified into two groups, the *α*-adrenergic family (comprising the *α*1 and *α*2 subtypes) and the *β*-adrenergic family (comprising the *β*1, *β*2, and *β*3 subtypes). NE exerts its action through either *α*1-, *α*2-, *β*1-, or *β*2-adrenoceptors in the central nervous system. *α*2-adrenoceptors located presynapticlly (autoreceptors) on the neural terminals inhibit NE release, whereas presynaptic *β*2-adrenoceptors enhance NE release upon activation. The release and effect of NE are modulated through interaction with these receptors. The action of NE in the synaptic cleft is ended largely through the neuron terminal reuptake by the NE transporter.

## Animal Model of 5-HT and NE in Somatic Symptoms

Rodent models of nociception demonstrate altered 5-HT and NE system function, and certain antidepressants enhance 5-HT and NE transmission. Both 5-HT and NE play a role in the descending inhibitory pathway, formed by projections descending from the brainstem or the midbrain to the spinal cord, which normally suppress painful inputs; thus, malfunction of these neurotransmitters may play a role in somatic syndromes, such as fibromyalgia and chronic headache (21).

It has been proposed that changes in 5-HT levels are part of pathophysiology of neuropathic pain. In mice deficient in 5-HT transporter (5-HTT-/- mice), the extracellular levels of 5-HT are increased in the brain; but the overall tissue concentrations of 5-HT are decreased. In contrast to wild-type mice, the 5-HTT-/- mice show absence of thermal hyperalgesia but present bilateral mechanical allodynia after an incomplete unilateral chronic constrictive injury of the sciatic nerve. The 5-HTT-/- mice also demonstrate higher levels of 5-HT in injured nerves and lower overall tissue levels of 5-HT than the wild-type mice (22, 23). Furthermore, the wild-type mice experience a longer period of thermal hyperalgesia and show higher levels of 5-HT in the sciatic nerves than the 5-HTT-/- mice after intra-plantar injection of Freund’s complete adjuvant (23). These findings suggest that 5-HT participates in nociception transmission and in reducing spinal inhibition in bilateral mechanical allodynia.

Using a rodent model of neuropathy induced by inflammatory mediator and nerve injury, Liu found that the intrathecal administration of the 5-HT1A receptor antagonist, rather than the 5-HT2 or 5-HT3 receptor antagonist, significantly attenuates the increased anti-nociception induced by the administration of morphine in intra-periaqueductal gray. Therefore, the 5-HT1A receptor is involved in the spinal descending inhibition pathway that suppresses nociception transmission in rats with nerve injury or inflammation (24). Abbott et al. found that the intra-plantar injection of 5-HT2A receptor antagonists may lead to peripheral analgesic effect (25). Another study found that neuropathic pain coincides with noradrenergic system disruption, as indicated by increased locus coeruleus bursting activity; enhanced expression of tyrosine hydroxylase, NE transporter, and *α*2 adrenergic receptors in the locus coeruleus; and hypersensitive *α*2 adrenergic receptors (26). Moreover, parathyroid hormone 2 receptor and TIP39 knockout mice display lower baseline nociceptive threshold and decreased inflammatory effect, and a blockade of *α*2-adrenoceptors could increase the thermal and tactile sensitivity in the knockout mice recovered from neuropathic injury (27). Antidepressant treatment of the SSRI fenfluramine was able to prevent mechanical allodynia, cold allodynia, and tonic pain in the model of neuropathic pain (28). Furthermore, treatment of venlafaxine (a serotonin-norepinephrine reuptake inhibitor (SNRI)), immediately after nerve injury, was able to inhibit the development of neuropathic pain; the antinociceptive effect of venlafaxine likely involves the *α*2-adrenoceptor (29).

Using a tail-flick rodent model, Eide et al. demonstrated that intrathecal injection of 5-HT1A and 5-HT1B receptor agonists could suppress the nociceptive tail-flick reflex at the spinal cord level, but none of them can change the temperature of tail skin (30). Dogrul et al. reported that the administration of the 5-HT7 receptor antagonist SB-269970 could inhibit the antinociceptive effect produced by systemic morphine administration, indicating that the spinal 5-HT7 receptor influences systemic morphine-induced antinociceptive actions (31). The authors also demonstrated that intrathecal injection of the 5-HT7 receptor antagonist abolishes the antinociceptive and antihyperalgesic effects produced by the systemic administration of paracetamol. Systemically administered paracetamol could stimulate 5-HT7 receptors at the spinal cord and activate descending serotonergic pathways (32).

Other animal models also suggest that somatic symptoms might be associated with impaired serotonergic and noradrenergic systems. In an exercise-induced chronic fatigue rat model, significantly increased levels of 5-HT and 5-HT transporter, decreased 5-HT1A mRNA expression (33), and significantly elevated 5-HT2A mRNA expression were found in various regions of the brain (34). Using an animal model with irritable bowel syndrome (IBS), a study found significantly upregulated expression of 5-HT7 receptor in the ileum and colon tissues compared with the control rats (35). Furthermore, treatment with 5-HT1A agonist/5-HT3 antagonist could inhibit the Bezold–Jarisch reflex and stress-induced defecation in this rat model; thus, agents that exert effects *via* 5-HT1A agonistic and/or 5-HT3 antagonistic activities might be beneficial for IBS treatment (36, 37). Another study also reported abnormal expression of colonic *α*2-adrenoceptors and NE reuptake transporter in different brain regions in a rat model of IBS (38).

## Neuroimaging Findings

Relatively few studies have examined 5-HT and NE system alterations in patients with somatic complaints using neuroimaging methods; published imaging studies primarily focused on 5-HT and NE receptors or 5-HT transporter and NET occupancies.

Several positron emission tomography (PET) studies have reported decreased 5-HT1A receptor binding in patients with panic disorder (39, 40), and the low 5-HT1A receptor binding may contribute to somatic symptoms associated with anxiety (41). Similarly, another PET study found a decrease in 5-HT1A binding in patients with chronic fatigue syndrome (42). Decreased 5-HT receptor binding may reflect a reduced number of 5-HT1A receptors or a decreased affinity of 5-HT or other ligands to the receptor. PET studies examining the relationship between 5-HT receptor/transporter binding and responses to noxious heat stimulation in healthy volunteers found a positive correlation between 5-HT2A binding and noxious stimulation (43). However, a negative correlation was observed between 5-HT transporter binding and response to tonic pain (44). Decreased 5-HT1A binding and changed 5-HT2A binding were detected in brain regions, including the hippocampus, amygdala, raphe nucleus, cingulate, insular cortex, prefrontal, parietal, temporal, and occipital cortices. These results suggest that the 5-HT neuronal function affects the activity of various brain regions. Abnormal 5-HT function in various brain regions may contribute to the development and modulation of somatic symptoms.

Shan et al. conducted a longitudinal MRI study to examine progressive brain changes in chronic fatigue syndrome. They found that white matter volumes in the left inferior fronto-occipital fasciculus was significantly reduced in patients with chronic fatigue syndrome (45). This result suggested that white matter abnormality in the inferior fronto-occipital fasciculus is associated with chronic fatigue syndrome. In addition, Chang et al. found subregions of the anterior cingulate cortex may play a role in the pathophysiology of chronic pain syndromes (46).

## Human Genetic Studies and Neuropharmacological Studies

Markoutsaki et al. reported an association between the population susceptibility of IBS and two single-nucleotide polymorphisms (SNPs) -1438 (G/A) and 102 (C/T) in the 5-HT2A receptor gene. They found that A allele and AA genotype of the -1438 (G/A) polymorphism in the 5-HT2A receptor gene show a significant association with IBS (47). Therefore, the carrier of A allele in this specific polymorphism in the 5-HT2A receptor gene might be a good candidate for IBS susceptibility.

Smith et al. conducted a study in 137 individuals that included patients with chronic fatigue syndrome, patients with mild fatigue, and those with no fatigue as controls. The study examined 77 polymorphisms in genes associated with 5-HT signaling (HTR1A, HTR1E, HTR2A, HTR2B, HTR2C, HTR3A, HTR3B, HTR4, HTR5A, HTR6, and HTR7), synthesis (TPH2), catabolism (MAOA), and transport (SLC6A4). Three biomarkers (-1438G/A, C102T, and rs1923884), located in the HTR2A gene of these polymorphisms examined, were identified to have a significant association with chronic fatigue syndrome. The HTR2A-1438 (rs6311) A allele, allele T of HTR2A C102T (rs6313), and C allele of HTR2A rs1923884 are more common in patients with chronic fatigue syndrome than in controls. Furthermore, silico analysis revealed that the A allele of -1438 is located in the core of the Th1/E47 consensus sequence and creates an allele-specific binding locus for neurodevelopment-associated transcription factor Th1/E47. These results indicate that polymorphism in the HTR2A gene is involved in the pathophysiology of chronic fatigue syndrome (48). A previous study also reported that the promoter activity in cells or tissues was higher in the A allele carrier of HTR2A-1438 (rs6311) than in the HTR2A G allele carrier. The authors suggest that HTR2A-1438 A polymorphism plays a role in promoter activity (49).

Felippotti et al. examined the role of noradrenergic mechanisms in the locus coeruleus in postictal antinociceptive effects. They microinjected yohimbine (an *α*2-receptor antagonist) and propranolol (a *β*-receptor antagonist) into the unilateral locus coeruleus and found that both yohimbine and propranolol injection to the locus coeruleus area caused a distinct decrease in antinociceptive effects. The blockade effect of yohimbine was more prominent compared with that of propranolol, possibly due to the presynaptically located *α*2-adrenoceptors in locus coeruleus neurons. These effects are associated with the noradrenergic regulation in locus coeruleus, suggesting that both *α*2- and *β*-adrenoceptors in locus coeruleus are involved in the mechanism underlying postictal antinociception (50).

## Clinical Therapeutics

Drugs used to treat somatic symptoms include antidepressants, antipsychotics, antiepileptics, and natural products, such as St. John’s wort (51). The effectiveness of these drugs has been reported by a limited number of studies (52–54). The proposed mechanisms include inhibition of spinal cord painful inputs, inhibition of prefrontal cortical areas that are involved in noxious activity, treatment of comorbid disease, and the direct effects on somatic symptoms.

Antidepressants are usually classified according to their impacts on neuronal synapses, such as inhibiting presynaptic transporters to block the reuptake of certain neurotransmitters, blocking certain neurotransmitter receptors, or the blockade of monoamine oxidase enzymes. Tricyclic antidepressants block the reuptake of NE and 5-HT neurotransmitters to achieve antidepressant therapeutic effects. However, tricyclic antidepressants also block M1, *α*1, and H1 receptors simultaneously, which can lead to diverse side effects, such as thirst, constipation, blurred vision, dizziness, orthostatic hypotension, sedation, lethargy, and weight gain in clinical applications. Other antidepressants include SSRIs such as fluoxetine, sertraline, paroxetine, and citalopram, SNRIs such as duloxetine and venlafaxine, and 5-HT receptor inhibitors such as mirtazapine. Evidence suggests that 5-HT and NE play an analgesic role in treating somatic symptoms through the spinal cord inhibitory descending pain pathway; however, their effects become aberrant in patients with somatic complaints (55–57). 5-HT and NE projection from brainstem descending the spinal cord could suppress painful inputs. The long-term administration of antidepressant treatments may enhance the efficacy of 5-HT synaptic transmission. Tricyclics enhance 5-HT synaptic transmission by increasing the sensitivity of postsynaptic 5-HT1A receptors, whereas SSRIs produce this effect by reducing the function of terminal 5-HT autoreceptors, thereby increasing the amount of 5-HT released. In addition, antidepressants may improve somatic symptoms, such as fatigue, anergy, or trouble sleeping, through their immunoregulatory effect (58–61).

The possible benefit of antipsychotics in somatic symptoms may be due to their analgesic effects (62), but the underlying mechanisms remains unclear. Their analgesic effect may be mediated by 5-HT antagonism (63), *α*2-adrenoreceptor stimulation (64), or other mechanisms. The mechanisms by which natural products such as St. John’s wort treat somatic symptoms also remain unclear. The efficacy of St. John’s wort on treating somatic complaints, including headache or gastrointestinal symptoms, is possibly secondary to the improvement in depression (65, 66). The effect of Hypericum extracts for somatic symptoms might be due to the inhibition of the reuptake of 5-HT, NE, and dopamine (67).

The application of antidepressants acting on 5-HT and NE systems for the treatment of somatic symptoms has been supported by many clinical trials and systematic reviews. For instance, a meta-analysis including 94 trials shows that antidepressants can substantially improve somatic symptoms (52). Another meta-analysis has shown that antidepressants appear to be effective in treatment of functional gastrointestinal disorders (68). In general, antidepressants have been used in the treatment of chronic pain syndromes, such as IBS (68, 69), chronic fatigue syndrome (70), fibromyalgia (71, 72), and other related somatic symptoms. Patients with fibromyalgia show low a threshold to pain that is caused by noxious stimuli, possibly due to the deficits in 5-HT and NE systems, which result in the failure to inhibit the painful inputs at the spinal cord level (73, 74). Jackson et al. summarized that antidepressants might be beneficial to treat 11 somatic symptoms including headache, chronic back pain, chronic facial pain, chronic pelvic pain, non-cardiac chest pain, fibromyalgia, IBS, tinnitus, chronic fatigue syndrome, interstitial cystitis, and menopausal symptoms (75).

Recent studies have found that the number of somatic symptoms in patients with depression who have not achieved remission show a significantly greater number of somatic symptoms than those who have achieved remission after 8 weeks of treatment with fluoxetine (76). Fluoxetine is also effective in improving somatic symptoms in adolescent patients with anxiety disorders and depression comorbid with severe somatic symptoms, such as stomachaches, restlessness, palpitations, blushing, sweating, muscle tension, and trembling/shaking (8). The relief of somatic symptoms may be due to the pharmacologic action to increase the levels of 5-HT in the synaptic cleft. In addition, another study reported that somatic symptoms markedly decreased in patients with depression after treatment with mirtazapine, a 5-HT receptor inhibitor (77). These results suggest that the 5-HT system dysfunctions are involved in the pathological mechanism of somatic symptoms.

Litoxetine is an antidepressant drug that combines 5-HT3 antagonism and 5-HT transporter inhibition to prevent the gastrointestinal and pain-augmenting side effects induced by SSRIs, such as sertraline (78). 5-HT3 receptor antagonists are effective in relieving symptoms, inhibiting urgency, and prolonging the transit of small and large bowel in IBS patients with diarrhea. However, agonists of 5-HT3 receptor was able to activate intestinal motility and shorten transit times in IBS patients with constipation (79). Revexepride, a 5-HT4 receptor agonist, could be a safe and effective candidate treatment for gastroparesis, a chronic gastric disorder characterized by clinical symptoms such as abdominal pain, vomiting, nausea, early satiety, postprandial fullness, and bloating (80).

Dolasetron, a 5-HT3 alternative inhibitor, is efficacious in the treatment of fibromyalgia (81). Administration of 5-HT3 receptor antagonists can significantly decrease pain intensity in patients with fibromyalgia and neuropathic pain (55). Thus, specific antagonism of 5-HT3 receptors is considered a possible treatment method for fibromyalgia, a condition characterized by chronic fatigue and pain (81).

Furthermore, several drugs acting on the 5-HT1A, 5-HT1B, and 5-HT1D receptors have been evaluated for their efficacy in treating migraine. Peroutka et al. demonstrated that migraine drugs, including ergotamine, dihydroergotamine, and sumatriptan, show affinity for the 5-HT1A, 5-HT1B, and 5-HT1D receptors, suggesting that these 5-HT receptors are involved in the action of the these antimigraine drugs (82, 83). Peroutka et al. summarized that acute antimigraine drugs, such as ergotamine and sumatriptan, show great affinity for 5-HT1D receptors and low affinity for 5-HT1A receptors. It has also been suggested that sumatriptan may not work through 5-HT1B receptors (84). These 5-HT receptors are located on certain intracranial blood vessels. In theory, the reduction of 5-HT may associate with the increased production of pain-inducing or vasoactive substances in the perivascular space, which may lead to angiectasis and migraine. 5-HT1D receptor agonists may facilitate 5-HT release and inhibit noxious stimulation (82). This phenomenon possibly explains the high prevalence of migraine in patients with depression.

Drugs acting on the noradrenergic system also have been implicated in the treatment of somatic symptoms. The SNRI duloxetine (60mg/day) can effectively reduce overall pain, shoulder pain, back pain, and time in pain while awake in patients with depression (85, 86). Another SNRI, venlafaxine, is also effective in improving neuropathic pain, as suggested by a randomized, double-blind, 10-week crossover trial (87). In addition, several double-blind, placebo-controlled trials reported that depressive patients with fatigue symptoms experienced overall improvement as well as remission in fatigue-related complaints following the treatment of levomilnacipran extended-release, a type of SNRI antidepressant. These results suggest that SNRIs are effective in the treatment of somatic symptoms (88).

Tricyclics, dual-acting antidepressants seem to be more effective than SSRIs in treating somatic symptoms. A meta-analysis suggested that tricyclics are superior to SSRI antidepressants in the therapy of various somatic symptoms, such as headache, idiopathic pain, fibromyalgia, tinnitus, irritable bowel disorder, and chronic fatigue in patients with chronic depression (52). Amitriptyline, the most studied tricyclic medication, is effective in treating at least one of the following complaints: pain, sleep, morning stiffness, overall improvement, fatigue, function symptoms, and tenderness. Desipramine predominantly inhibits the reuptake of NE and, to a minor extent, inhibits the reuptake of 5-HT. Dinan et al. suggested that treatment of desipramine may alleviate IBS by blocking the abnormal function of central *α*2 noradrenergic receptors (89). Tricyclic antidepressants are effective in treating somatic symptoms, possibly because of their ability to block the reuptake of 5-HT and NE.

## 5-HT and NE Interaction

Substantial interactions exist between the serotonergic and noradrenergic systems in the central nervous system. Both 5-HT neurons and noradrenergic neurons are active and affect each other in the locus coeruleus (90–92). In addition, the serotonergic system interacts with other neurotransmitter systems such as dopaminergic inputs from the midbrain corpus striatum (93) and glutamatergic and inhibitory γ-aminobutyric acid-ergic inputs from forebrain regions (94) and local interneurons (95–97).

Projections from 5-HT neurons to NE neurons are inhibitory. For instance, rats with damage in 5-HT neurons show a greater firing activity of NE neurons than intact animals (98). Previous studies also demonstrated that long-term administration with SSRIs might increase 5-HT transmission, presumably increasing the effectiveness of 5-HT projections to locus ceruleus and forebrain neurons (99). For instance, Szabo et al. found that the short-term administration of citalopram exerts no effect on the firing activity of NE neurons; however, the long-term treatment of citalopram could produce a progressive reduction of the spontaneous firing activity of NE neurons (100).

Other evidence suggests that the interaction between NE transporter (NET182C) and 5-HT transporter (5-HTTLPR) polymorphisms is associated with susceptibility and electroconvulsive therapy treating response in antidepressant treatment resistant depression patients. Patients with combined NET and 5-HT transporter polymorphism genotypes had poorer treatment responses (101). Moreover, functional and structural interactions with NE, 5-HT and dopamine systems that are known to have an impact on executive control processes (102, 103). Furthermore, researchers observed interactions between 5-HT transporter and a functional NET polymorphism, suggesting 5-HT and NE interplay in shaping goal-directed behavior (103, 104). Most interestingly, interactions of 5-HT transporter and NET polymorphism also influence cognitive and executive functioning, such as target accuracy and event-related potential, latency in n-back task (105).

In addition, studies have shown that mirtazapine can significantly increase the firing of 5-HT neurons and trigger a small but distinct increase in the firing of NE neurons (106, 107). Behavioral tests suggest that depletion of NE might block the effects of some SSRIs as well (108). These results have provided evidence that antidepressants selectively working on the serotonergic system may also indirectly influence the function of the noradrenergic system. In addition, blockade of the 5-HT2A receptor may potentiate the release of NE under the treatment of SSRI (109).

## Conclusion

Somatic symptoms are highly prevalent in patients with depression, anxiety and some other psychiatric disorders. In this narrative review, we examined the potential role of serotonergic and noradrenergic systems in the development and treatment of various somatic symptoms. Antidepressants may play an important role in the therapy of somatic symptoms by regulating 5-HT and NE neurotransmitter systems at central and peripheral levels. Future research combining neuroimaging techniques and genetic analysis to further elucidate the biological mechanisms of somatic symptoms and to develop novel treatment strategies is needed.

## Author Contributions

All authors listed have made a substantial, direct and intellectual contribution to the work and approved it for publication.

## Funding

This study was supported by grants from the National Key R&D Program of China (2016YFC1307100 and 2016YFC1306900) and the National Natural Science Foundation of China (Grant Nos. 81571310, 81630033, 81771447, and 81471363).

## Conflict of Interest Statement

The authors declare that the research was conducted in the absence of any commercial or financial relationships that could be construed as a potential conflict of interest.
